# Evaluating the Experiences of Occupational Therapists and Children Using the SensoGrip Pressure-Sensitive Pen in a Handwriting Intervention: Multimethods Study

**DOI:** 10.2196/51116

**Published:** 2024-03-07

**Authors:** Lena Rettinger, Erna Schönthaler, Andrea Kerschbaumer, Carina Hauser, Carissa Klupper, Lea Aichinger, Franz Werner

**Affiliations:** 1 Health Assisting Engineering FH Campus Wien, University of Applied Sciences Vienna Austria; 2 Occupational Therapy FH Campus Wien, University of Applied Sciences Vienna Austria

**Keywords:** handwriting, handwriting pressure, pen, children, occupational therapy, assistive technology, tablet, app

## Abstract

**Background:**

The acquisition of handwriting skills is essential for a child’s academic success, self-confidence, and general school performance. Nevertheless, an estimated 5% to 27% of children face handwriting challenges, where the ability to modulate pressure on the pencil and lead on the paper is a key motor component.

**Objective:**

We aimed to investigate the experience with and usability of the SensoGrip system, a pressure-measuring pen system with personalized real-time feedback about pressure modulation, in a clinical setting with children and occupational therapists (OTs).

**Methods:**

A multimethods study was conducted, incorporating qualitative interviews and questionnaires with children, user diaries, focus group discussions, and a usability questionnaire with OTs, along with a questionnaire for parents.

**Results:**

The study involved OTs (n=8), children with handwriting difficulties (n=16), and their parents (n=16), each of whom used the SensoGrip system in up to 5 therapy sessions. OTs reported that the SensoGrip system helped to focus the child’s awareness on handwriting pressure and to measure it objectively. The system received high acceptance and usability ratings from the OTs—usefulness: median score of 4 out of 7; ease of use and ease of learning: median score of 6 out of 7; and satisfaction: median score of 6 out of 7. Participants appreciated that it fosters pressure awareness and motivation to draw and write.

**Conclusions:**

The SensoGrip pressure-sensing system with real-time feedback is a promising tool for pediatric occupational therapy. It supports children with handwriting difficulties to adjust their pressure application during the task. In the future, controlled quantitative trials are warranted to further examine the system’s impact.

## Introduction

### Background

The development of handwriting skills is not only important for building children’s self-confidence but is also considered a fundamental element for academic success [1,2] and educational achievement [3]. Numerous studies have indicated that many children encounter challenges in acquiring handwriting skills. According to a review by Hartingsveldt et al [4], the prevalence of handwriting problems ranges from 5% to 27%. Handwriting is a multifaceted task that requires the integration of motor, sensory, perceptual, praxis, and cognitive functions [5,6]. An essential motor aspect involves the precise control of pencil pressure and pressure of the lead on the paper, as excessive pressure on the pen when writing can cause muscle fatigue. Children with handwriting problems have less capacity for idea generation, planning, and revision when they have to focus on the handwriting mechanics [7]. The aim of teachers and occupational therapists (OTs) is that children obtain readable, fluent, and efficient individual handwriting without becoming tired [8]. A survey of 2000 German teachers revealed that sustained writing was a problem for >60% of children in elementary or secondary school, most often based on handwriting-associated cramps (73%) and incorrect pencil grip (68%) [8]. Lin et al [9] observed that children exhibit difficulties in pressure adjustment when learning graphomotor skills. Previous studies have already measured grip or tip pressure (pressure of the pen on the writing surface) using a pen with built-in sensors [10,11]. However, these systems were built for research purposes only. There is a need to investigate the role of pressure in pencil use in a natural setting and to provide direct feedback mechanisms for the children. Biofeedback is a method for changing unconscious movements and perceptions into conscious ones and has already been used in the context of a handwriting training device by the company, “Schneider,” and their pen, “Base Senso.” Biofeedback is known to be effective in the treatment of many musculoskeletal conditions and has been shown to, for example, improve the measures of balance and patients’ exercise techniques [12].

However, to the best of our knowledge, currently, there is no tool that records the child’s pressure and provides individualized feedback to the child and OT. Further limitations of the currently commercially available technologies include the following: very high acquisition costs; insufficient calibration accuracy; usability issues, as training is required to use the app; incomplete recording of key measurement parameters; and lack of feedback [13].

### The SensoGrip Project

The SensoGrip project was launched with the aim of creating a pressure-sensitive pen, focusing on user-centered conception, development, and evaluation. Previously, we had conducted a comprehensive evaluation to understand the needs of all relevant stakeholders, steering the further development process [14]. The project was supported by an interdisciplinary team that included professionals from occupational therapy, physical therapy, special education, medical informatics, computer science, and mechanical engineering. We adopted an iterative development process complemented by simultaneous testing phases to continuously refine the features.

### The SensoGrip System

**Figure 1:**
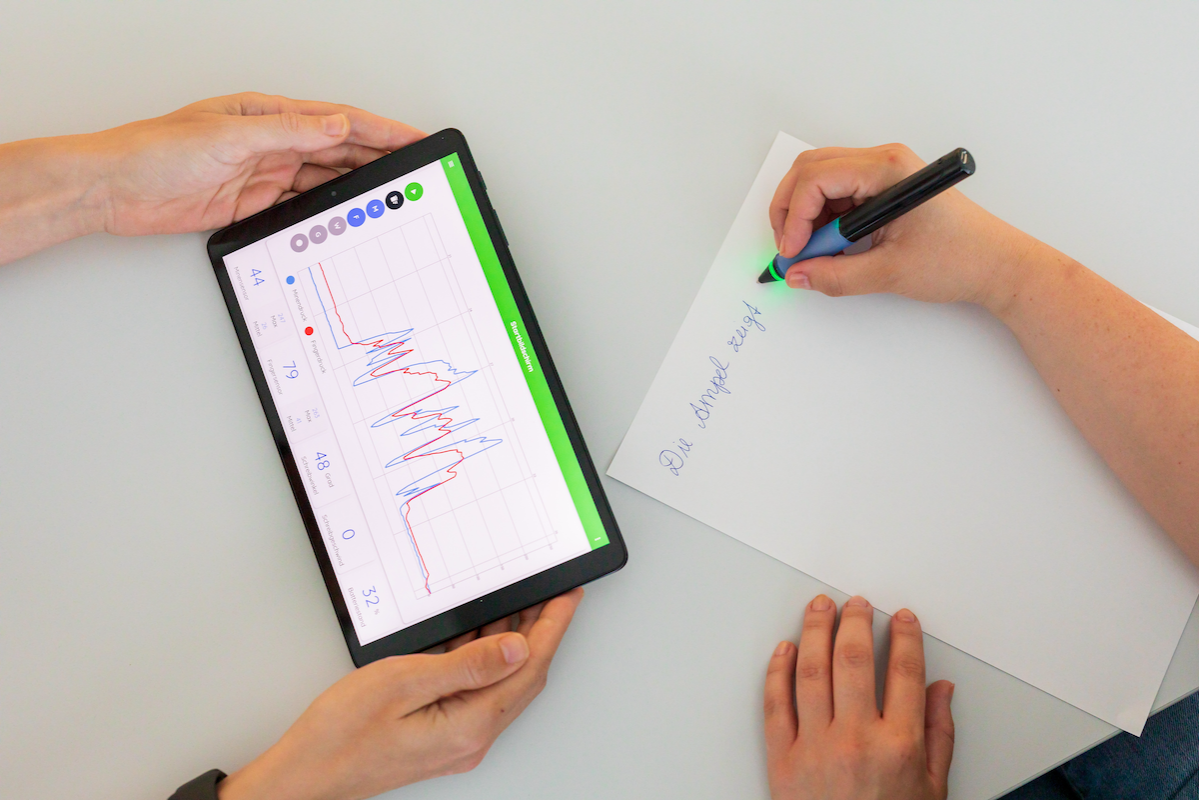
The SensoGrip pen with activated feedback LED and the SensoGrip app with line graphs for grip pressure (red) and tip pressure (blue).

The SensoGrip system consists of 2 components: a smart SensoGrip pen and the SensoGrip mobile app. The pen weighs 24 g, is 140 mm long and 14 mm in diameter, and has a roller pen refill (Figure 1).

The SensoGrip pen contains 2 sensors to measure the pressure applied on the grip area (grip pressure) and the pressure applied by the pen on the paper (tip pressure) respectively. An LED ring is placed between the distal end of the grip area and the pen tip. The LED provides visual feedback about the applied pressures according to the individual settings in the mobile app. The battery of the pen can be recharged using a standard micro-USB cable.

**Figure 2:**
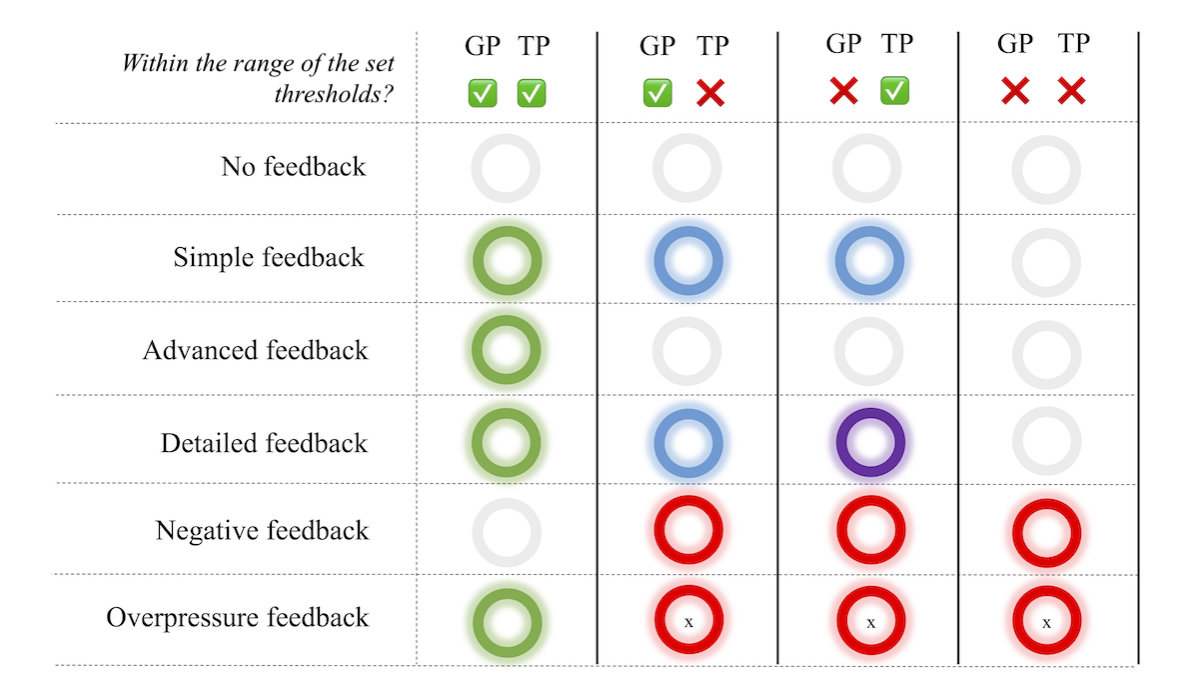
Feedback modes offered by the SensoGrip system. Depending on the mode selected in the SensoGrip app, the LED ring of the SensoGrip pen lights up in individually chosen colors. GP: grip pressure; TP: tip pressure; x: only if pressure is very high.

The SensoGrip app runs on the Android operating system on a customary tablet. It allows for the creation of customer profiles with individual settings and displays real-time or recorded measurements. On the basis of the individual needs and preferences of the child, different feedback modes can be chosen (Figure 2). Upper and lower thresholds are set by the OT to choose the pressure range within which the selected feedback is displayed by the LED. The thresholds are set for the grip pressure and tip pressure separately. Colors for different feedback modes can be chosen individually.

The app provides real-time visualization of pressure data through numerical displays and line graphs for both grip and tip pressures, as illustrated in Figure 1. Users have the option to capture these data alongside a video of the writing hand in action. For ease of analysis, the app allows the display of customizable threshold lines on the graphs, which can be toggled on or off as needed. All recorded data remain retrievable for future reference. In addition, the interface supports the simultaneous comparison of graphs from different sessions. For reporting or further analysis, users can export these data directly into a PDF document.

### Aim

This study is part of a pilot study involving a single-case experimental design [15] to assess the effectiveness of the system. The findings concerning the effectiveness of the system, as derived from the Single-Case Experimental Design study, will be discussed in a subsequent publication. The study was registered on ClinicalTrials.gov (NCT05014854). The aim of this paper was to present data about the usability, acceptance, and perceived impact of the SensoGrip system.

The following research questions were used to guide this study:

1. How is the usability characterized?
2. What hurdles exist in the actual use of the individual components?
3. How are the acceptance factors of the system evaluated by the target groups?
4. What is the perceived impact of the system?
5. What are the intended and unintended effects of the system on the target groups?
6. Does the system positively influence children’s motivation and adherence?

## Methods

### Overview and Procedure

The study was conducted between July 2021 and October 2021 in Vienna and Lower Austria, Austria, across various private practices of OTs. Each participating child engaged in 3 to 7 therapy sessions, during which the OTs integrated the SensoGrip system into therapy. OTs received comprehensive training from the research team. This training included a range of essential skills, such as operating the SensoGrip system, creating patient profiles, fine-tuning feedback settings, interpreting the graphical representation of pressure data, and familiarizing themselves with the procedures for assessment and data upload. Although OTs were expected to incorporate the SensoGrip system into every therapy session for a minimum of 10 minutes, they were granted the flexibility to use it more extensively as needed. The research team supplied a manual containing a variety of recommended therapeutic activities tailored to the SensoGrip system. Moreover, the OTs were empowered to personalize the system settings, including the calibration of pressure thresholds for both finger and tip feedback and the selection of feedback types and colors. The integrity and consistency of the intervention’s implementation were carefully tracked through the collection of user diaries, analysis of use data from the SensoGrip pens, and evaluations conducted during posttherapy focus groups and interviews.

**Figure 3:**
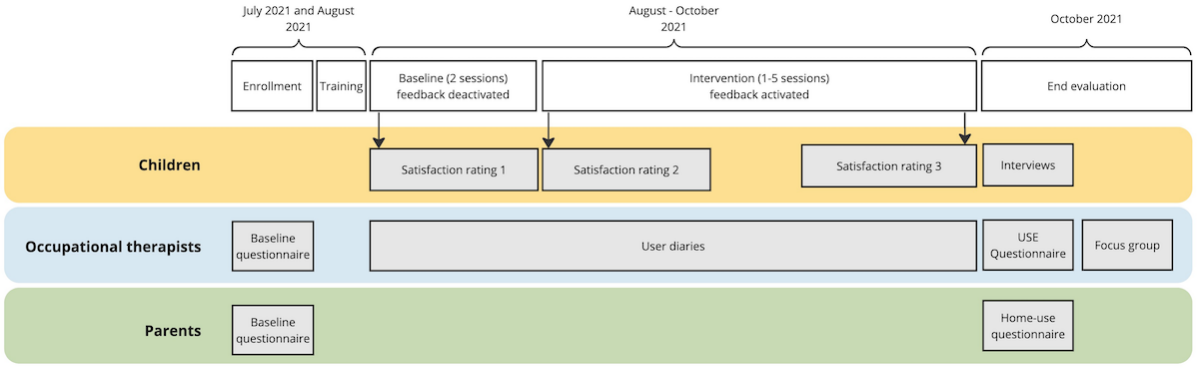
Study overview and timeline. USE: Usefulness, Satisfaction, and Ease of Use and Ease of Learning.

To assess the usability and user acceptance of the SensoGrip system and to gain early insights into the perceived impacts of use, a multimethods design was implemented (Figure 3).

To obtain a comprehensive understanding of the SensoGrip system’s impact, we included a variety of data collection methods. We conducted qualitative interviews with children and captured their satisfaction with a child-friendly smiley rating scale. OTs provided baseline data about the child’s handwriting issues; regularly documented SensoGrip use, their observations, and the systems’ performance in user diaries; rated its usability using a questionnaire; and participated in a focus group. Parents provided information about the child’s handwriting at home through a baseline questionnaire. They also described their experiences with the SensoGrip system when it was used in the home setting. The methods were chosen carefully to meet the respective needs of the study participants in terms of time, effort, and place and to achieve a combination of qualitative and quantitative results for triangulation.

### Participants

#### Recruitment and Enrollment Procedure

Participants were recruited using the snowball sampling technique [16], in which initial contacts with OTs in private practices were established through multichannel outreach. This included distributing emails to all pediatric OTs registered in the region, engaging with OT-specific Facebook groups, and leveraging the personal networks of the project team. In addition, OTs were encouraged to use their professional and social networks to further distribute participant invitations. We structured participation into teams or dyads composed of an OT and ≥1 children under their care, with the option to involve the children’s parents or legal guardians. Inclusion in the study was contingent upon meeting the established criteria, and upon indicating interest, OTs were provided with detailed participation checklists and consent documentation. Once eligibility was confirmed and consent was obtained, OTs, their paired children, and the children’s legal guardians were formally enrolled in the study.

#### Children

Children aged between 5 and 10 years and exhibiting difficulties in handwriting, especially in handwriting pressure adjustment were eligible. Children belonging to this age group were selected as the target group because they are in the developmental period during which children typically acquire foundational handwriting skills. OTs assessed the eligibility based on a handwriting pressure checklist, where at least 2 stated criteria had to be present. The checklist contained 6 indicators of excessive writing pressure, 4 indicators of insufficient writing pressure, and 1 criterion for high fluctuations in writing pressure (Multimedia Appendix 1). Children had to be able to follow verbal instructions and maintain attention in graphomotor activities for at least 10 minutes and had to have adequate emotional regulation and age-appropriate psychosocial skills. Children who were not able to hold a pen, owing to stiffened joints or excessive or insufficient muscle tension, could not participate in the study. Children’s eligibility to participate in the study was assessed by their individual OT, who then selected children for the study from their patient group. Before starting the assessment and intervention, children and parents (or legal guardians) signed an informed consent form.

#### Occupational Therapists

OTs were eligible to participate if they had at least 2 years of professional experience in evaluating and treating graphomotor difficulties in children. In addition, they had to provide occupation-based therapeutic services aimed at addressing handwriting challenges. OTs were not eligible if they rejected using technical tools in therapy or stated that they are not used to handling everyday technologies such as smartphones. For a collaborative dyad to be formed within the study, each participating OT was required to enlist at least 1 child from their clinical practice. Informed consent was mandatory; OTs were required to sign an informed consent form before enrolling in the study.

#### Parents or Legal Guardians

Parents or legal guardians were eligible to participate if their child consented to use the SensoGrip system at home between therapy sessions. A prerequisite for participation was proficiency in basic, everyday technology use. Informed consent was obtained before their inclusion in the study.

### Assessments

A comprehensive set of tools was used to collect both qualitative and quantitative feedback from OTs, children, and their parents.

#### User Diaries (OTs)

OTs maintained a user diary to record the use of the SensoGrip system, experiences and thoughts about the system, and issues with its usability and functionality. These recordings were a central element, as they allowed to observe several therapy sessions of each child retrospectively without directly participating in the sessions themselves. After each session of use, the OTs self-assessed to check whether any technical issues occurred (yes or no and which?), whether the feedback felt reasonable (yes or no and why?), whether they found the SensoGrip system useful (yes or no and why?), whether the system was intuitive to use (yes or no and why?), and how much they enjoyed using it (5-point Likert scale). In addition, the OTs maintained notes about how the SensoGrip system was integrated into the therapy session. The diary was developed by the project team, and the understandability and quality were assessed along with an OT before starting the trial.

#### Usability Questionnaire (OTs)

At the end of the intervention period, the usability of the SensoGrip system was assessed by the OTs via the standardized Usefulness, Satisfaction, and Ease of Use and Ease of Learning (USE) questionnaire [17,18], translated into German by the research team (Multimedia Appendix 2). It consists of 30 items, attributed to dimensions such as usefulness, ease of use, ease of learning, and satisfaction, which are rated on a 7-point Likert scale (1=do not agree at all; 7=totally agree).

#### Smiley Rating Scale (Children)

Children self-assessed their satisfaction with the SensoGrip system using a 6-point smiley rating scale. Children were asked “How much did you enjoy writing with the SensoGrip pen?” in the first therapy session of the intervention, in which feedback from the pen was deactivated to not influence the baseline measurements for the single-case experimental design study, and in the first therapy session in which feedback was activated. After the final session, they were asked, “How good can you write with the SensoGrip pen?” and “How much do you like the SensoGrip pen?”

#### Questionnaires (Parents)

Before initiating the study, parents or legal guardians were asked to complete a detailed questionnaire designed to understand the child’s handwriting practices at home. It covered several topics, including the frequency and duration of writing activities at home, handwriting legibility, pressure and speed during writing, challenges encountered, and the acceptance and use of tools for writing and learning, along with any related social and emotional concerns. Furthermore, when the SensoGrip pen was used at home between therapy sessions, parents or legal guardians provided end-of-study feedback through a subsequent questionnaire. This follow-up sought to assess their perceptions about the pen’s effectiveness, user-friendliness, and overall impact in the home environment.

#### Interviews (Children)

After the intervention, child participants were interviewed individually by 2 experienced team members, both women, with a background in pediatric occupational therapy. These interviews were deliberately scheduled immediately following the final therapy session at the OT’s office to mitigate any additional stress for the children, a particularly vulnerable group. Parents or legal guardians were allowed to attend the interview, if this was deemed beneficial. The semistructured interviews (Multimedia Appendix 3), which were pretested with age-matched children, explored a range of topics: the children’s enjoyment in using technical tools in general, their previous experience with handwriting, their evaluation of the SensoGrip system’s functionality, the advantages they perceived from its use, their willingness to continue using the system, their suggestions for its improvement, and their 3 most and least effective aspects. The interviews were audio recorded and varied in duration between 10 and 30 minutes per child. In an effort to minimize any potential discomfort, the children were not asked to confirm the accuracy of the interview content.

#### Focus Group (OTs)

OTs participated in a structured focus group interview designed to elicit a comprehensive evaluation of their experiences with the SensoGrip system. The choice of focus group format was intentional; it was selected for its capacity to yield nuanced insights through collective discussions among the OTs. The focus group was facilitated by 2 experienced research team members with a background in pediatric occupational therapy. To ensure a setting that minimized distractions, the focus group was conducted in a quiet meeting room at the university and lasted 108 minutes. An additional researcher documented field notes to capture nonverbal behaviors and observations. The semistructured guide (Multimedia Appendix 3) included open-ended questions along with prompts and probes and covered the following topics: prevalence of handwriting difficulties and, especially, handwriting pressure difficulties in praxis; common concepts and methods for addressing those issues; integration of the SensoGrip system into OT praxis; perceived benefits and barriers when using the SensoGrip system; effects of pressure feedback about children’s handwriting and behavior; ease of learning the SensoGrip system; assessment of the SensoGrip system regarding design and functionality; and suggested improvements for SensoGrip pen and app. The guideline was developed by the research team. A pilot test was not conducted, but the questions were intensively discussed within the team to ensure that the research questions were addressed. If an OT was unable to attend the focus group owing to scheduling conflicts, an individual interview was conducted. This ensured comprehensive inclusion of their insights regarding the SensoGrip system. Consistent with the focus group methodology, this interview adhered to the established guidelines and was audio recorded to capture the OT’s feedback accurately. In contrast, the focus group session was video recorded, allowing for precise attribution of comments to the respective contributors. Subsequently, the findings from the study were shared in a public forum, and all the involved OTs were encouraged to attend. This presentation served as an opportunity for participant validation, where OTs could review and comment on the reported results—a process known as member checking.

### Data Analysis

#### Questionnaires and User Diaries

User diary data were systematically compiled into an Excel (Microsoft Corporation) spreadsheet, enabling a detailed analysis of the technical and usability challenges encountered during the SensoGrip system’s operation. Statistical analysis included the calculation of the median and the minimum and maximum scores from the children’s smiley rating scale. Similarly, we computed the median values for the usability ratings derived from the USE questionnaire’s subscales. The frequency distributions of these ratings, along with the smiley rating scale scores, were then visually represented through graphical illustrations.

#### Qualitative Data

Content analysis based on the procedure suggested by Kuckartz [19] was performed on completely verbatim transcripts of the focus group and interviews by 2 researchers using the software, MAXQDA 2022 (VERBI Software, 2021). This method allows a combination of deductive and inductive coding. Deductive codes were based on the topics that guided the interviews: functionality, stability, usefulness, usability, ease of learning, barriers, performance expectancy, effort expectancy, social influence, hedonic motivation, facilitating conditions, intention to use, effect on handwriting pressure, transfer into daily living, effect on motivation and adherence, effect on therapeutic efficiency, and support in documentation. Then, inductive codes were differentiated into many subtopics such as design, usability, and barriers. The 2 researchers collaborated intensively in the coding and analysis phases to increase objectivity. Working in tandem, they cross-examined each other’s coding decisions and interpretations during the analysis and discussed discrepancies to reach consensus. This approach aimed to reduce individual bias and enhance the reliability of the findings.

### Ethical Considerations

The SensoGrip system is defined as a class-1 active medical device according to Rule 12 of Directive 93/42/EEC [20]. Therefore, the evaluation of the system qualified as a clinical trial and was successfully approved by the ethics committee of the City of Vienna under the number EK-21-042-0321. In addition, the study was registered at the Austrian Federal Office for Safety in Health Care [21] as required by national law. The study was monitored on an ongoing basis by a physician and a monitor. No adverse effects occurred.

## Results

### Description of Participants

Overall, 8 OTs (n=7, 88% women; n=1, 13% men) participated in the study. They were aged between 28 and 51 (mean 37.6, SD 7) years and had between 4 and 30 (mean 13.5, SD 8.2) years of experience in pediatric occupational therapy. All (8/8, 100%) used a smartphone or mobile tablet with 3 to 5 apps (4/8, 50%) or >5 apps (4/8, 50%) on a regular basis. The participating OTs’ acceptance of technology was rather high (Multimedia Appendix 4).

Overall, 16 children (n=3, 19% girls; n=13, 81% boys) were enrolled in the study (Table 1). They were aged between 5 and 10 years. Of the 16 children, 14 (88%) wrote with their right hand, 1 (6%) wrote with the left hand, and 1 (6%) did not have a preferred hand for writing at the time of the study. Their reasons for referral to OT were developmental coordination disorder of fine and gross motor coordination, unspecified developmental disorder of motor function, difficulties in concentration, dyspraxia, sensory integration disorder, autism spectrum disorder, and adaptive disorder.

Of the 16 parents, 9 (56%) reported that their child’s hand grew tired when writing, 7 (44%) reported that their child had to shake their hand for relaxation when writing, and 1 (6%) reported that their child verbalized pain regularly when writing. Of the 16 parents, 10 (63%) thought that fatigue had an influence on the handwriting of their child, 9 (56%) found prolonged writing to be a relevant factor, 7 (44%) perceived that the pen their child was using influenced the handwriting, and 1 (6%) mentioned that time pressure negatively affected handwriting. Of the 16 parents, 4 (25%) rated their child’s handwriting as illegible, 2 (13%) as sloppy, and 1 (6%) as often smudgy. Of the 16 children, 8 (50%) had trouble in maintaining alignment with the line when writing, 3 (19%) imprinted their handwriting on the next page, and 4 (25%) produced very large letters when writing. Of the 16 children, 8 (50%) used special aids for writing such as grip aids with or without molds, weighted writing utensils, or special ergonomic pens. Of the 16 parents, only 2 (13%) confirmed that the aids were helpful. Of the 16 children, 4 (25%) enjoyed their use and 1 (6%) explicitly did not like it. Of the 16 parents, 5 (31%) acknowledged that handwriting problems frequently led to conflicts at home.

**Table 1:** Overview of children’s baseline data.

Child’s ID	Sex	Age	Handedness
C1	Male	6 y and 11 mo	Right
C2	Male	6 y and 6 mo	Right
C3	Male	7 y and 8 mo	Right
C4	Male	6 y and 6 mo	Right
C5	Male	9 y and 4 mo	Right
C6	Male	6 y and 0 mo	Right
C7	Male	7 y and 8 mo	Right
C8	Male	5 y and 10 mo	Left
C9	Male	5 y and 9 mo	Right
C10	Male	6 y and 9 mo	No preference
C11	Male	5 y and 8 mo	Right
C12	Female	9 y and 3 mo	Right
C13	Female	10 y and 11 mo	Right
C14	Male	6 y and 2 mo	Right
C15	Male	8 y and 4 mo	Right
C16	Female	8 y and 5 mo	Right

### Relevance of Handwriting Pressure in OT Practice

According to the participating OTs, the prevalence of handwriting problems among children in their common practice is approximately 30%, and one-third of these children also shows signs of inappropriate handwriting pressure. Problems of handwriting pressure adjustment rarely occur in isolation; they occur in combination with other difficulties related to handwriting grip and letter formation. OTs select therapy approaches to target appropriate handwriting pressure adjustment that include activities to improve body perception in general and occupation-based activities such as drawing and writing with different materials. Common activities mentioned were coloring by hatching with varying intensity or applying padding of varying modalities under the paper. All OTs emphasized that they used a child-centered approach in terms of child-initiated color or topic selection.

### Application of the SensoGrip System in the Study

Of the 16 children, 12 (75%) used the SensoGrip system in 5 therapy sessions, 3 (19%) used it in 3 sessions, and 1 (6%) used it only in 1 therapy session. On average the total use time was 77 (SD 34; range 10-135) minutes per child. Reasons for discontinuation of implementing the SensoGrip system were based on unforeseen therapy termination (1/16, 6%) or the child’s pencil grip being very immature (2/16, 13%). The children used the SensoGrip system in a variety of writing and drawing exercises, ranging from playful activities to more structured tasks such as free drawing, tracing, copying, and writing. OTs supported the children in monitoring the feedback from the LED indicator on the pen and in adjusting the pressure on the pen and paper. In addition, the accompanying mobile app was introduced, offering an interactive experience where they engaged in creating specific graph patterns. By varying the pressure on the pen, children learned to manipulate the graphical representations, striving to achieve either high or low pressure readings or to maintain consistent pressure levels. OTs reviewed the children’s handwriting pressure with them, using the graphical data recorded in the mobile app after various writing and drawing activities. In a home setting, 31% (5/16) of the children continued to use SensoGrip between therapy sessions. According to the parents of these 5 children, 1 (20%) child used it daily, 2 (40%) used it multiple times per week, and 2 (40%) used in weekly. Some OTs opted not to send the SensoGrip pen home owing to concerns about potential loss or damage or worries that the pen might not be used as intended or returned for subsequent sessions.

### OTs’ Evaluation

**Figure 4:**
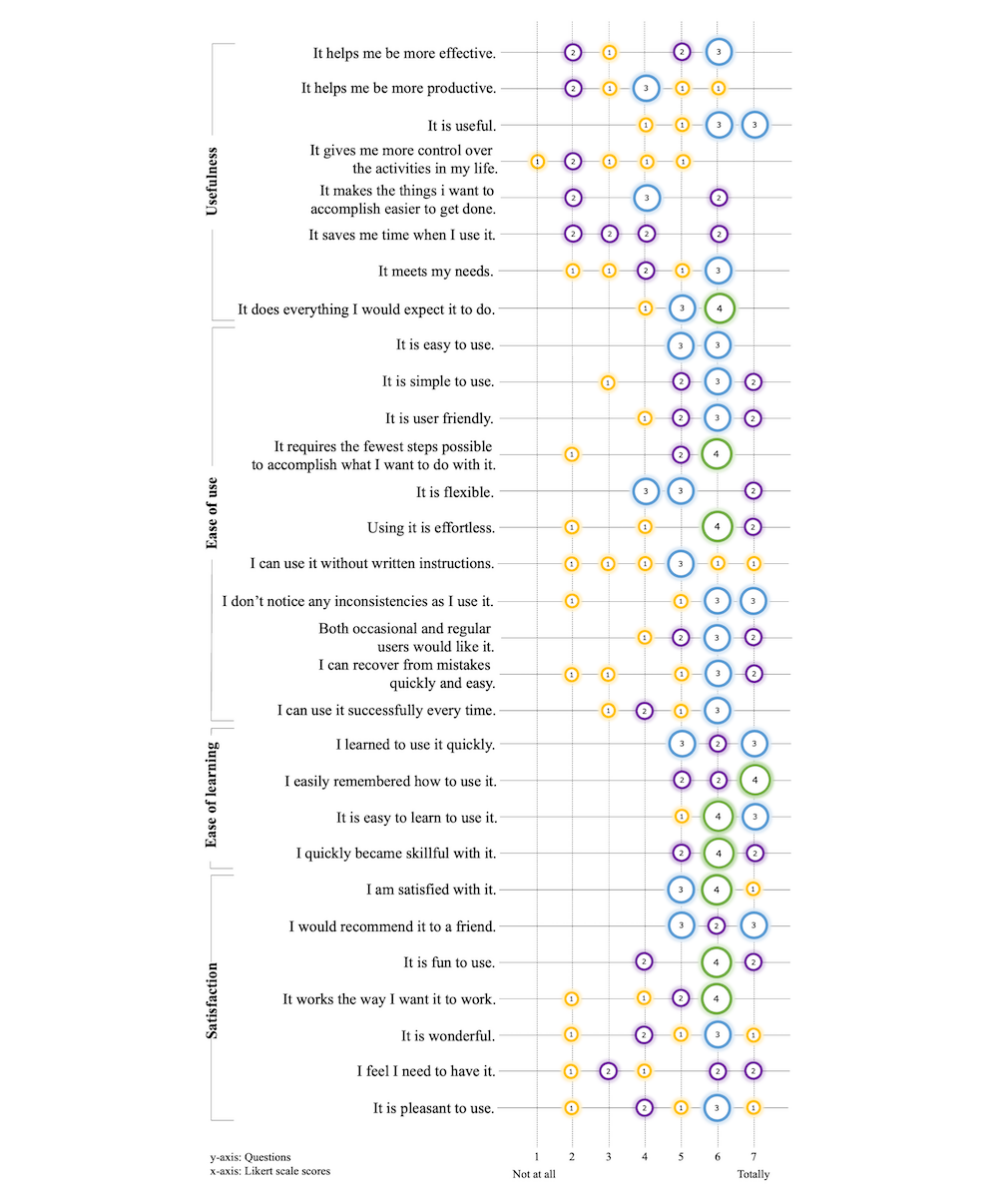
Ratings of the Usefulness, Satisfaction, and Ease of Use and Ease of Learning questionnaire. Bubbles indicate the number of participants who rated the respective score for the respective question. Missing numbers indicate skipped questions.

Regarding the USE questionnaire’s usefulness subscale, OTs reported a median score of 4 (IQR 3-6) out of 7, indicating a moderate level of perceived utility of the SensoGrip system (Figure 4). During the focus group discussions, OTs gave high ratings to the tablet’s graphical representation of handwriting pressure, valuing it as a particularly useful tool for objectively assessing a child’s performance and informing therapeutic strategies. They noted the advantages of the system’s real-time visual pressure feedback, which was well received by both OTs and children alike. OTs also expressed appreciation for the customizable settings, which allowed them to tailor the feedback to each child’s specific requirements. A notable benefit reported was the SensoGrip pen’s utility in the home environment, where children could continue practicing even when the OT was not present:

I think it is great when they take it home. You just set everything up and say, for example, “This week try to make it light up as much as possible when you do your homework.”

OT 3

OTs assigned high ratings to the SensoGrip system’s ease of use (median 6, IQR 5-6) and ease of learning (median 6, IQR 6-7), each receiving a median score of 6 out of 7 on a Likert scale, which suggests a high level of usability of the system (Figure 4). They found the graphical analysis of pressure to be intuitive to use and the customization to be straightforward. However, determining the optimal thresholds for each child using the graphical interface proved challenging for some. An OT expressed a preference for adjustment based on numerical pressure values rather than graphical data. To further improve the system’s usability, the OTs recommended enhancements, such as ensuring the mobile app’s functionality even when the pen is not connected or is charging. This would facilitate uninterrupted access to settings and data. They also proposed a feature to provide isolated feedback about either the finger or tip pressure, which would allow a focused approach to correcting specific pressure issues. Further suggestions included more sophisticated data comparison tools, such as visualizations showing the duration for which a child maintains pressure within the set thresholds and box plot analysis. In addition, a filtering function to extract particular data points was suggested. For future iterations, OTs advocated for the development of an automated progress analysis feature and integration of interactive games into the SensoGrip mobile app to enrich the SensoGrip experience.

The OTs provided a median score of 6 (IQR 5-6) on the satisfaction subscale of the USE questionnaire on a 7-point Likert scale (Figure 4). They pointed out that although they had stated many suggestions for improvement, they would like to use the SensoGrip system in its current development state:

On the other hand, if it would be possible to buy this pen, I would do it.... It is actually a good product.

OT 5

It is really usable the way it is.

OT 2

Overall, the OTs noted that the use of the SensoGrip system helped to focus the child’s awareness on handwriting pressure and to measure it objectively. An OT expressed that the system helped to identify the specific situations in which the handwriting pressure increased. OTs perceived improvement in handwriting pressure in some children, based on observation. Nevertheless, some children did not benefit from the system. OTs hypothesized that differences in impact might depend on the age of the children:

It was my impression that the older child, which is in the first grade, improved it’s handwriting pressure. His problem was that he was holding the pen too loosely. And now it is more adequate, and the tracing became better. The younger children’s handwriting pressure did not really change.

OT 4

### Children’s Evaluation

During the interview, 69% (9/13) of the children mentioned that they thought the SensoGrip system was useful. They reported an increased awareness of their handwriting pressure when using the SensoGrip pen, which they felt contributed positively to their writing:

It really helps me figure things out. Like, when the pen lights up, I know “oh, the pressure is very low here.”

C15; aged 8 y

When I do it right, the light turns green. And when I push too hard, then it turns purple.

C13; aged 10 y

When I push very hard and then soft, the line goes up and down. Then again harder and softer, and so on.

C3; aged 7 y

Other children did not perceive any differences when writing with the SensoGrip pen or preferred using their normal pen:

No, not necessarily. I can still write better with a pencil.

C14; aged 6 y

**Figure 5:**
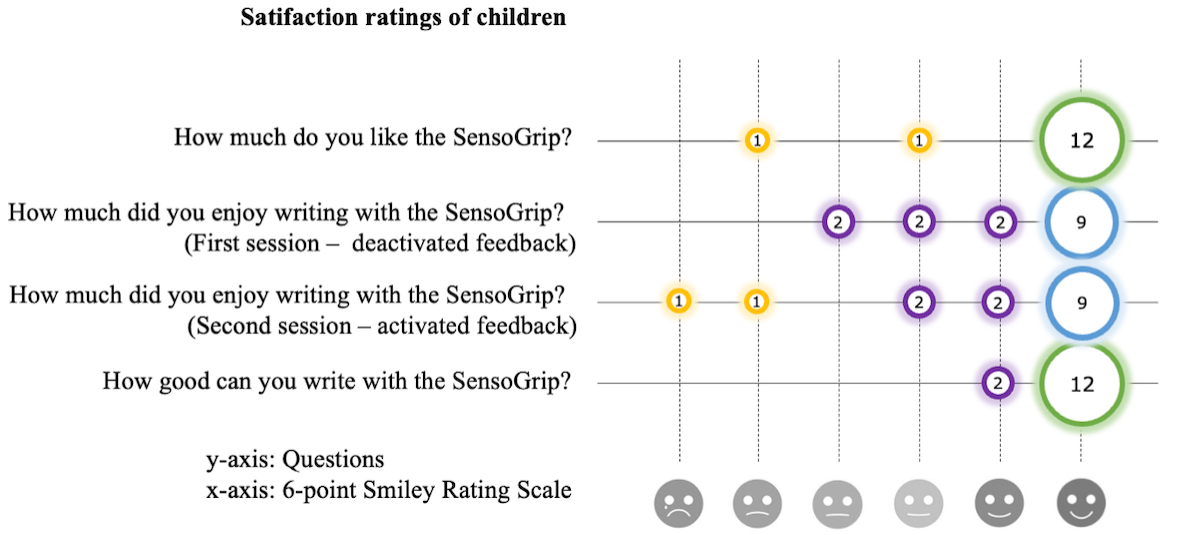
Children’s satisfaction ratings on a 6-point smiley rating scale. Bubbles indicate the number of children who rated the respective smiley for the respective question. Missing numbers indicate missing answers.

Children assessed their satisfaction with the SensoGrip system using the smiley rating scale (Figure 5).

Overall, 80% (12/15) of the children gave the highest possible rating when asked how much they like the SensoGrip system. Furthermore, 86% (12/14) of the children rated the question, “How good can you write with the SensoGrip pen?” with the highest score (Likert scale score=6), and 14% (2/14) of the children rated with the second highest score (Likert scale score=5). In the interviews, they explained that it was “quite easy to write with the SensoGrip (pen)” (child 2 and child 3; aged 6 y) and that it was “easy to hold” (child 15; aged 8 y). However, some children encountered issues when using the SensoGrip pen: a child mentioned that they had trouble keeping the LED light on (child 15; aged 8 y), a child reported that the ink stained their fingers (child 14; aged 6 y), a child found the LED light not sufficiently bright (child 3; aged 6 y), and another child had difficulties in maintaining a firm grip on the pen (child 3; aged 7 y).

Overall, children reported a medium to high level of enjoyment when using the SensoGrip system. The median rating was 6 (4.25-6) on a 6-point Likert scale (minimum=1; maximum=6), where 6 represents maximum writing enjoyment (Figure 5).

Overall, 92% (12/13) of the children thought that the SensoGrip pen was “cool” or “fun,” and 75% (9/12) of them said that they would enjoy continuing to write with the SensoGrip pen. Only 8% (1/13) of the children mentioned that the feedback puts them “out of control” and that it would not help them with writing (child 14; aged 6 y). They most enjoyed the LED feedback, the sensor technology, and working with the app’s graph:

...That we could draw hills in the app. And the colored light. And that it was so pleasant for my fingers. That were my three favorites.

C3; aged 7 y

...I could see if I am doing it right.

C13; aged 10 y

...It feels good in my hands. The light. The feedback. And that it helped me with writing.

C1; aged 6 y

Some children expressed improvement in writing during the interviews:

Now I can write much better.

C1; aged 6 y

Earlier I pushed the pen a little harder on the paper and I can see that it is now different.

C13; aged 10 y

My hand felt a little bit lighter when I was holding the pen like this.

C15; aged 8 y

### Parents’ Evaluation

Among the 5 parents who had the SensoGrip pen used at home, 3 (60%) found the SensoGrip pen to be intuitive or rather intuitive in its use, whereas 1 (20%) felt that it was not intuitive. Overall, among the 5 parents, 2 (40%) were satisfied with the SensoGrip pen, 2 (40%) were neutral about it, and 1 (20%) did not respond. Of the 5 parents, 3 (60%) were in favor of continuing its use, 1 (20%) opted against it, and 1 (20%) did not respond to this specific question.

### Participants’ Design Evaluation

Participants evaluated the pen’s design based on various features, as described in Table 2.

**Table 2:** Opinions about the different design features of the SensoGrip pen. Occupational therapists (OTs) and children’s opinions were obtained from the interviews, and parents’ ratings were obtained using the questionnaire.

Design feature	Opinions		
	Children	Parents	OTs
Overall appearance and design	“Good” (12/13, 92%) “Medium” (C14; aged 6 y)	Very suitable: 2/5, 40% Suitable: 3/5, 60%	—^a^
Size and weight	“Good” (C3; aged 6 y) “Heavier than a conventional pen but great” (C15; aged 8 y) “Should be a little bit thinner” (C1; aged 6 y)	Size Very suitable: 1/5, 20% Suitable: 2/5, 40% Indifferent: 1/5, 20% Not suitable: 1/5, 20% Comments—Too thick (2/5, 40%) Weight Suitable: 3/5, 60% Mediocre: 2/5, 40% Shape Suitable: 3/5, 60% Indifferent: 2/5, 40%	“Okay, but could be smaller, thinner, and lighter for better fit for children. Pen’s tip could be a little bit shorter.”
Material and haptics	“Pleasant” (C3; aged 6 y) “It can be held well” (C1; aged 6 y)	Very suitable: 2/5, 40% Suitable: 3/5, 60%	“Anti-slip surface was good. Grip moulds could help some children to ensure ergonomic grip.”
Finger sensor position	—	—	“For some children hard to position fingers on the sensor, to ensure correct pressure measurements. Sensor should be placed nearer towards the pen’s tip.”
LED position	—	LED should be positioned on the proximal end of the pen (1/5, 20%)	“LED should be positioned on the proximal end of the pen for younger children (ensures better sight of the LED) and on the distal end for older children (ensures simultaneous sight of LED and written text).”
LED	“Funny when it lights up” (C15; aged 8 y) “Not bright enough” (C3; aged 6 y)	Colored LED motivated children (3/5, 60%), but also distracted one child (1/5, 20%) Wish for acoustic feedback (2/5, 40%)	“Should be brighter. Some wished additional acoustic and/or vibration feedback.”
Pen’s tip and refill	“Well slipping pen tip” (C3; aged 6 y)	Tip runs smoothly on the paper (3/5, 60%) Ink not erasable (2/5, 40%) Pencil lead would be better (3/5, 60%)	“Pencil lead would be better for younger children, colored pencil lead even better. Roller pen ink should be erasable.”
Battery	—	Runs down too fast (2/5, 40%) Battery display missing (1/5, 20%) “Poor” battery (1/5, 20%)	—

^a^Not available.

### Technical Performance

Overall, the SensoGrip pen and app were found to be technically well functioning. The reported malfunctioning included the following: quick battery depletion and a long time to connect the pen to the app in some cases. Of the 16 SensoGrip pens, 2 (13%) broke. In one case, it fell on the floor, and in another case, a child was applying extremely high pressure on the pen. In one instance, the lead of the pen slipped inside the pen when a child was pressing it with very high pressure on the table. Crashing of the tablet app was reported only once over the test duration.

## Discussion

### Usefulness, Satisfaction, and Perceived Impact of the System

OTs viewed the SensoGrip system as a valuable addition to their therapeutic toolkit. It met or exceeded the expectations for most, with 7 out of 8 (88%) OTs rating it highly on the USE questionnaire for its usefulness. The system’s graphical display of writing pressure was particularly noted for its effectiveness in analyzing and guiding children’s handwriting interventions. In addition, some children reported improvements in their handwriting, attributing this to the heightened pressure awareness provided by the biofeedback. This tool seems to provide information about sensory-motor processes during writing, which are not inherently perceptible to them [22].

Overall, the OTs were pleased with the system’s performance, finding it enjoyable and effective—a sentiment that remained consistent throughout several weeks of therapy. This consistent satisfaction is indicative of the system’s potential for long-term acceptance, avoiding the pitfall of waning interest over time [23].

Children’s satisfaction was also noteworthy, with almost all (12/15, 80%) expressing the highest level of enjoyment. The interactive feature of the pen lighting up was a favorite. However, caution was advised for children with intellectual impairments, as a child’s difficulty in comprehending the feedback suggested the need for tailored use assessments by OTs, especially given the possible correlation between intellectual and graphomotor challenges.

In summary, the SensoGrip system was recognized for its dual impact: enhancing awareness of handwriting pressure and increasing children’s motivation to engage in writing tasks during therapy sessions.

### Usability and Technical Performance

The SensoGrip system earned high scores for user-friendliness from OTs, with a median score of 6 out of 7 on the Likert scale. The ease with which users could learn the system was also rated highly, with scores ranging between 5 and 7. Feedback about future refinements included a preference for a thinner, lighter pen—a sentiment echoed by some children and parents. However, current design limitations prevent the reduction of the pen’s thickness. In addition, the OTs suggested shortening the pen’s tip to allow the child’s hand to be closer to the paper while still keeping the fingers on the pressure-sensing zone on the grip area.

The OTs reported that most children easily adapted to writing with the SensoGrip pen. There was a consideration to reposition the LED to the pen’s proximal end for better visibility for the OT, but the need for children to see the light during writing mandated its placement near the tip. A preference for pencil lead over ballpoint refills was noted, particularly for young children accustomed to pencils. The prototype’s design accommodated a fixed-length ballpoint refill to avoid the complexities associated with a retracting pencil lead and pressure measurement.

Technical performance evaluations throughout the trial revealed that the system functioned at a high level. Most recorded technical issues during the trial were generally minor and typical for technical products, such as battery depletion and slow app response. The only significant technical issue occurred when 2 pens broke owing to falling on the ground and excessive pressure, which was attributed to the limitations of the manufacturing process in which the pen shafts were 3D printed. Despite these incidents, overall technical performance was not deemed to significantly influence user satisfaction or the system’s usability.

### Limitations

This study has certain limitations. The selection of OTs was based on their readiness to integrate a technical device into their practice, which may not reflect the perspectives of those with low technical proficiency. Consequently, the findings may predominantly represent the views of OTs who are already inclined toward technology, suggesting a potential bias toward perceiving the system as having considerable potential. This limits the broad applicability of the results across the entire OT population. Children’s overwhelmingly positive feedback about the pen must be considered in light of possible bias, as responses might have been influenced by the desire to provide socially acceptable answers to adults. In addition, the study was conducted within the same institution responsible for developing the SensoGrip system. However, the study’s integrity was maintained by ensuring that the research team was different from the development team. Given the primarily qualitative and explorative nature of the study and the absence of a control group, the findings reflect the subjective experiences of the participants. As such, the reported impacts should be interpreted with an understanding that they do not provide an empirical measure of the system’s effectiveness.

### Conclusions

This multimethods study evaluating the SensoGrip pressure-sensitive pen system offers insightful contributions to the field of pediatric occupational therapy. Through the involvement of 8 OTs with varying levels of experience (mean 13.5, SD 7 y); 16 children aged between 5 and 10 years, exhibiting handwriting difficulties; and their parents, the study describes the system’s utility and potential. The participants engaged with the SensoGrip system within a natural, private practice therapy setting in Austria.

Our findings reveal that the SensoGrip system is met with strong acceptance and satisfaction, both from children who enjoyed the interactive feedback and from OTs who recognized its potential as a therapeutic tool. The system was instrumental in enhancing the children’s awareness of handwriting pressure, thus showing the potential to promote more controlled and deliberate movements. OTs reported observing tangible improvement in the children’s pressure modulation over the course of the intervention, which included 3 to 7 therapy sessions. However, the SensoGrip system’s suitability varied among participants, with a subset of children not experiencing the anticipated benefits. These variances highlight the need for personalized approaches in the application of assistive technologies within pediatric occupational therapy.

The study underscores the importance of such assistive technologies in reinforcing the development of fine motor skills. In particular, the real-time feedback component of the SensoGrip system was highlighted as a significant motivator for children, fostering both engagement and enjoyment in the handwriting process.

Although the SensoGrip system has shown promising results in this preliminary exploration, future studies involving controlled quantitative trials are essential to validate and quantify its impact. This study will ideally expand to consider the effects of age, developmental stage, and presence of comorbid conditions on the efficacy of the SensoGrip system.

The feedback from both the children and OTs underscore the potential of integrating technology-based interventions in therapeutic settings. Such interventions contribute not only to skill development but also to the intrinsic motivation of children, which is crucial for sustained engagement and therapeutic success.

## Appendix

Multimedia Appendix 1 Eligibility checklist for children, assessed by their occupational therapist.

Multimedia Appendix 2 Usefulness, Satisfaction, and Ease of Use and Ease of Learning questionnaire, German translation.

Multimedia Appendix 3 Focus group and interview guidelines.

Multimedia Appendix 4 The participating therapists’ acceptance of technology.

## Abbreviations

OT: occupational therapist
USE: Usefulness, Satisfaction, and Ease of Use and Ease of Learning
