# Supplementary material for: Evaluating the Experiences of Occupational Therapists and Children Using the SensoGrip Pressure-Sensitive Pen in a Handwriting Intervention: Multimethods Study
Source: JMIR Rehabil Assist Technol. 2024 Mar 7;11:e51116. doi: 10.2196/51116 (PMC10958334; doi:10.2196/51116)
Supplement: Multimedia Appendix 1 [file rehab_v11i1e51116_app1.pdf]

Eligibility checklist for children, assessed by their occupational therapist. At least two criteria had to apply.

1) The child complains about pain when writing/drawing.
2) The child’s finger knuckles turn white, due to high grip pressure when writing/drawing.
3) The distal interphalangeal joint of the index and/or middle finger is severely hyperextended when writing/drawing.
4) The writing/drawing imprints on the next page.
5) The child must take regular breaks when writing/drawing in order to relax the hand.
6) The child's fingers slide to the tip of the pencil when writing/drawing because the pencil is held too loosely
7) The child's writing/drawing is difficult to see because too little pressure is being applied
8) The pen slips out of the child's hand when writing/drawing
9) The child cannot guide the pencil sufficiently because it is held too loosely
10) The child frequently breaks the pencil mine while writing or drawing or pushes a felt-tip pen into the pencil.
11) Grip pressure or pressure on the paper varies greatly during writing/drawing
