# Supplementary material for: Evaluating the Experiences of Occupational Therapists and Children Using the SensoGrip Pressure-Sensitive Pen in a Handwriting Intervention: Multimethods Study
Source: JMIR Rehabil Assist Technol. 2024 Mar 7;11:e51116. doi: 10.2196/51116 (PMC10958334; doi:10.2196/51116)
Supplement: Multimedia Appendix 2 [file rehab_v11i1e51116_app2.pdf]

USE Fragebogen*

Es folgen nun einige Aussagen zu Stift und Tablet-App. Geben Sie bitte anhand der Skala von 1 bis 7 an, wie sehr sie den Aussagen zustimmen. Denken Sie nicht lange über Ihre Antwort nach und behalten Sie im Hinterkopf: Es gibt keine richtigen oder falschen Antworten. Nur Ihre Meinung.

	**Nützlichkeit**		**1**	**2**	**3**	**4**	**5**	**6**	**7**		**keine Antwort**	**Kommen-tare**
1.	Es hilft mir, effektiver zu sein.	**stimme gar nicht zu**	**O**	**O**	**O**	**O**	**O**	**O**	**O**	**stimme absolut zu**		
2.	Es hilft mir, produktiver zu sein.	**stimme gar nicht zu**	**O**	**O**	**O**	**O**	**O**	**O**	**O**	**stimme absolut zu**		
3.	Es ist nützlich.	**stimme gar nicht zu**	**O**	**O**	**O**	**O**	**O**	**O**	**O**	**stimme absolut zu**		
4.	Es gibt mir mehr Kontrolle über die Aktivitäten in meinem Leben.	**stimme gar nicht zu**	**O**	**O**	**O**	**O**	**O**	**O**	**O**	**stimme absolut zu**		
5.	Es erleichtert das Erledigen der Dinge, die ich erreichen möchte.	**stimme gar nicht zu**	**O**	**O**	**O**	**O**	**O**	**O**	**O**	**stimme absolut zu**		
6.	Es spart mir Zeit, wenn ich es verwende.	**stimme gar nicht zu**	**O**	**O**	**O**	**O**	**O**	**O**	**O**	**stimme absolut zu**		
7.	Es entspricht meinen Bedürfnissen.	**stimme gar nicht zu**	**O**	**O**	**O**	**O**	**O**	**O**	**O**	**stimme absolut zu**		
8.	Es macht alles, was ich davon erwarte.	**stimme gar nicht zu**	**O**	**O**	**O**	**O**	**O**	**O**	**O**	**stimme absolut zu**		
	**Benutzerfreundlichkeit**											
9.	Es ist einfach zu verwenden.	**stimme gar nicht zu**	**O**	**O**	**O**	**O**	**O**	**O**	**O**	**stimme absolut zu**		
10.	Es ist unkompliziert zu verwenden.	**stimme gar nicht zu**	**O**	**O**	**O**	**O**	**O**	**O**	**O**	**stimme absolut zu**		
11.	Es ist benutzerfreundlich.	**stimme gar nicht zu**	**O**	**O**	**O**	**O**	**O**	**O**	**O**	**stimme absolut zu**		
12.	Es erfordert wenige Schritte um das zu erreichen, was ich damit tun will.	**stimme gar nicht zu**	**O**	**O**	**O**	**O**	**O**	**O**	**O**	**stimme absolut zu**		
13.	Es ist flexibel.	**stimme gar nicht zu**	**O**	**O**	**O**	**O**	**O**	**O**	**O**	**stimme absolut zu**		
14.	Es zu verwenden ist mühelos.	**stimme gar nicht zu**	**O**	**O**	**O**	**O**	**O**	**O**	**O**	**stimme absolut zu**		
15.	Ich kann es ohne Gebrauchsanweisung verwenden.	**stimme gar nicht zu**	**O**	**O**	**O**	**O**	**O**	**O**	**O**	**stimme absolut zu**		
16.	Ich bemerke keine Widersprüchlichkeiten wenn ich es benutze.	**stimme gar nicht zu**	**O**	**O**	**O**	**O**	**O**	**O**	**O**	**stimme absolut zu**		

17.	Sowohl gelegentlichen als auch regelmäßigen Nutzern würde es gefallen.	**stimme gar nicht zu**	**O**	**O**	**O**	**O**	**O**	**O**	**O**	**stimme absolut zu**		
18.	Bei Fehlern kann ich einfach und schnell wieder zurück.	**stimme gar nicht zu**	**O**	**O**	**O**	**O**	**O**	**O**	**O**	**stimme absolut zu**		
19.	Ich kann es immer erfolgreich benutzen.	**stimme gar nicht zu**	**O**	**O**	**O**	**O**	**O**	**O**	**O**	**stimme absolut zu**		
	**Einfaches Erlernen**											
20.	Ich habe schnell gelernt, es zu verwenden.	**stimme gar nicht zu**	**O**	**O**	**O**	**O**	**O**	**O**	**O**	**stimme absolut zu**		
21.	Ich erinnere mich leicht daran, wie man es benutzt.	**stimme gar nicht zu**	**O**	**O**	**O**	**O**	**O**	**O**	**O**	**stimme absolut zu**		
22.	Es ist einfach die Nutzung zu erlernen	**stimme gar nicht zu**	**O**	**O**	**O**	**O**	**O**	**O**	**O**	**stimme absolut zu**		
23.	Ich wurde schnell geschickt im Umgang damit.	**stimme gar nicht zu**	**O**	**O**	**O**	**O**	**O**	**O**	**O**	**stimme absolut zu**		
	**Zufriedenheit**											
24.	Ich bin damit zufrieden.	**stimme gar nicht zu**	**O**	**O**	**O**	**O**	**O**	**O**	**O**	**stimme absolut zu**		
25.	Ich würde es einem Freund/einer Freundin empfehlen.	**stimme gar nicht zu**	**O**	**O**	**O**	**O**	**O**	**O**	**O**	**stimme absolut zu**		
26.	Es macht Spaß, es zu benutzen.	**stimme gar nicht zu**	**O**	**O**	**O**	**O**	**O**	**O**	**O**	**stimme absolut zu**		
27.	Es funktioniert so, wie ich das haben möchte.	**stimme gar nicht zu**	**O**	**O**	**O**	**O**	**O**	**O**	**O**	**stimme absolut zu**		
28.	Es ist wunderbar.	**stimme gar nicht zu**	**O**	**O**	**O**	**O**	**O**	**O**	**O**	**stimme absolut zu**		
29.	Ich glaube, ich muss es haben.	**stimme gar nicht zu**	**O**	**O**	**O**	**O**	**O**	**O**	**O**	**stimme absolut zu**		
30.	Es ist angenehm es zu verwenden.	**stimme gar nicht zu**	**O**	**O**	**O**	**O**	**O**	**O**	**O**	**stimme absolut zu**		

**Zählen Sie die negativsten Aspekte auf:**
1.
2.
3.

**Zählen Sie die positivsten Aspekte auf:**
1.
2.
3.

* Ins Deutsche übersetzt aus: Lund, A. M. (2001). Measuring usability with the use questionnaire. *Usability interface*, *8*(2), 3–6.
