# Supplementary material for: Evaluating the Experiences of Occupational Therapists and Children Using the SensoGrip Pressure-Sensitive Pen in a Handwriting Intervention: Multimethods Study
Source: JMIR Rehabil Assist Technol. 2024 Mar 7;11:e51116. doi: 10.2196/51116 (PMC10958334; doi:10.2196/51116)
Supplement: Multimedia Appendix 3 [file rehab_v11i1e51116_app3.pdf]

Focus group guideline (Occupational therapists)

How often do you see children with graphomotor problems, especially with difficulties in pressure dosage?

Please briefly describe how you worked with children with pressure dosage difficulties before the study.

What changes has SensoGrip brought to your therapeutic approach?

- How was it used?
- What changed in the process?
- Time spent?

What impact did working with SensoGrip have?

- Curiosity
- Motivation
- Improvements/Deteriorations in pressure dosage
- Visual appearance of handwriting
- Writing duration
- Distraction by feedback
- Efficiency

How did the children handle the feedback?

How was your experience learning to use SensoGrip?

- Settings
- Evaluation and interpretation of data

What are your thoughts on the current prototype and its design?

- Shape
- Size
- Weight
- Material
- Design

What would you change about the prototype?

What would you change about the app?

What do you consider to be the 3 most positive and the 3 most negative features of SensoGrip?

Is there anything else you would like to mention?

Interview guideline (Children)

Which technical devices do you use in everyday life?
Tablet, computer, smartphone?

Describe how it feels for you when you draw or write for a longer period!

How do you like the SensoGrip pen? What do you think about ...? How could … be improved?
- Weight
- Size
- Shape
- Material
- Appearance

How has the pen helped you?
- Is writing easier?
- Are you more motivated?
- Is your handwriting better?

Do you write differently now than before?
What have you noticed?
What have others said (therapist, parents, teachers)?

Remember writing with the SensoGrip
- What was easy?
- What was difficult?
- What was fun? (e.g., certain exercises...)
- What was not?

How long did it take you to learn how to write properly with the SensoGrip pen?

- Did you know right away how to write with the pen, or did someone have to explain it to you?

Did your therapist let you look at the tablet?
What did you try with the tablet?
What could you see there?

How well did the pen work?
Was it sometimes broken?

Who have you told about the SensoGrip pen?
- What did they say?

If the pen was used at home: What did you use the SensoGrip pen for at home?
Drawing, homework, other
How often?

Would you like to continue using the SG?
- If yes, why?
- If no, why not?

Tell me 3 great and 3 not-so-great things about the SG!
