# Supplementary figures and images for: Evaluating the Experiences of Occupational Therapists and Children Using the SensoGrip Pressure-Sensitive Pen in a Handwriting Intervention: Multimethods Study
Source: JMIR Rehabil Assist Technol. 2024 Mar 7;11:e51116. doi: 10.2196/51116 (PMC10958334; doi:10.2196/51116)

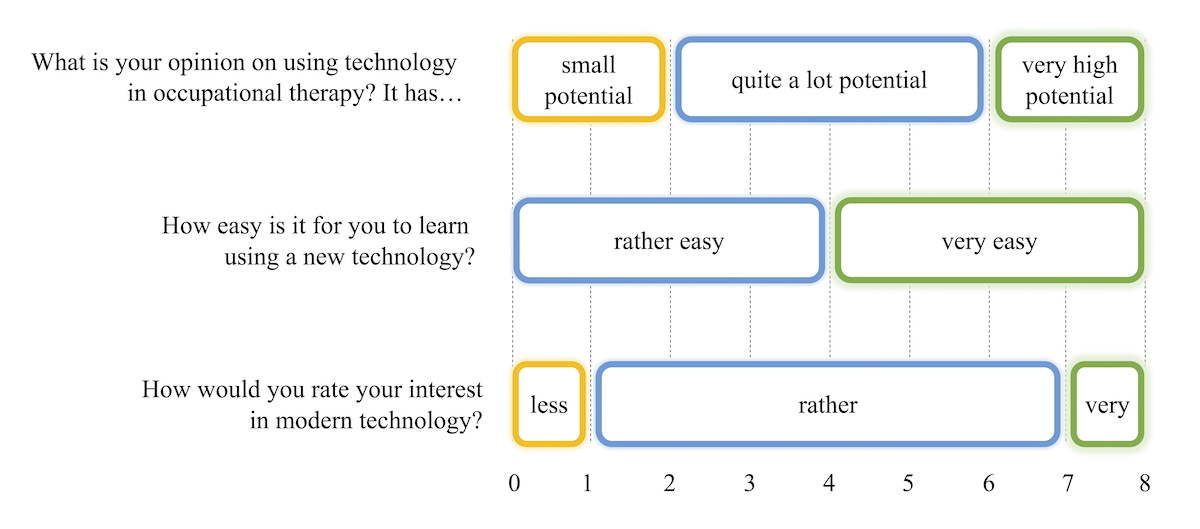

Supplement: Multimedia Appendix 4 [file rehab_v11i1e51116_app4.png]
